# The Growth Behavior for Intermetallic Compounds at the Interface of Aluminum-Steel Weld Joint

**DOI:** 10.3390/ma15103563

**Published:** 2022-05-16

**Authors:** Xiaoquan Yu, Jiankang Huang, Tao Yang, Ding Fan

**Affiliations:** 1 School of Materials Science and Engineering, Lanzhou University of Technology, Lanzhou 730050, China; yuxiaoquangood@163.com; 2 State Key Laboratory of Advanced Processing and Recycling Non-Ferrous Metals, Lanzhou University of Technology, Lanzhou 730050, China; liu_2310@163.com

**Keywords:** Al-Fe intermetallic compounds, growth kinetics, aluminum-steel weld joint, numerical simulation

## Abstract

In this work, the microstructure and growth behavior of Al-Fe intermetallic compounds (IMCs), which formed at interface of weld steel-aluminum joint, are successfully analyzed via the combination of experiment and physical model. A layer IMCs consists of Fe_2_Al_5_ and Fe_4_Al_13_, in which the Fe_2_Al_5_ is the main compound in the layer. The IMCs layer thickness increases with the increase of the heat input and the maximum thickness of IMCs layer is 22 ± 2 μm. The high vacancy concentration of Fe_2_Al_5_ IMCs provides the diffusion path for Al atoms to migrate through the IMCs layer for growing towards to steel substrate. By using the calculated temperature profiles as inputs, the combined 2D cellular automata (CA)-Monte Carlo (MC) model is applied to simulate the grain distribution and interfacial morphology evolution at the Al-steel interface. This 2D model simulates the IMCs nucleation, growth, and solute redistribution. The numerical results are in good agreement with the experimental results, suggesting that the growth process can be divided four stages, and the thickness of the Fe_2_Al_5_ layer increases nonlinearly with the increase of the growth time. The whole nucleation and growth process experienced 1.7~2 s, and the fastest growth rate is 8 μm/s. The addition of Si element will influence diffusion path of Al atom to form different interface morphology. The effects of peak temperature, cooling time, and the thermal gradient on the IMCs thickness are discussed. It shows that the peak temperature has the major influence on the IMCs thickness.

## 1. Introduction

Aluminum-steel dissimilar weld joints are widely used in the automotive industry because of it can achieve a lightweight design, which has advantages of energy saving and emission reduction [1]. However, joining Al to steel is a challenge due to some large differences between thermal-physical properties, including melting point, thermal conductivity, and coefficient of thermal expansion [2]. A brittle Al-Fe intermetallic compounds (IMCs) layer will be formed at steel and aluminum interface, which can lead to deleterious effect on the mechanical properties. It has been reported that the thickness, chemical composition, and morphology of the Al-Fe IMCs layer plays an important role in determining the mechanical strength of Al-Fe weld joint [3].

Various investigations have been proposed to control the IMCs layer from heat input aspect, including some new welding processes, such as, using a MIG-TIG double-sided arc welding-brazing process to join aluminum and steel, Ye et al. [4] reported that this method could achieve a thinner IMCs layer of 2.3 μm than conventional MIG process due to double side gas shielding. Marya et al. [5] developed a laser-roll method to join steel to aluminum, a narrow and ductile Fe_3_Al and FeAl IMCs layer was formed due to a low interfacial heating. With respect to the relationship between heat input and IMC layer, Wang et al. [6] investigated the effects of heat input on the IMC layer thickness as well as tensile strength, they found that the increasing linear energy brought some new IMCs phases which led to the formation of microcracks. Xue et al. [7] reported that a higher heat input resulted in a thicker and new morphology of Fe_4_Al_13_ IMCs layer, and the IMCs phase with a needle-like structure was helpful to improve the tensile strength of weld joint. Meco et al. [8] showed that the laser power density and interaction time determined the thickness of IMCs layer in the laser welding process, and the laser power will have negative effect on the bond strength when it over a certain threshold.

In addition to controlling heat input, one also can change the IMCs phases via the alloy metallurgy method by using filler wire or powder. Using addition of alloy elements to control the IMCs phases, Furuya et al. [9] found that the addition of Si and Ti elements could reduce the thickness of IMCs layer, and Ni, Cr, Ti, and Mn elements would cause a grain refinement of Fe_2_Al_5_ phase. Both the thin IMCs layer and fine grain IMCs show an effective enhancement of the joint strength. To change the chemical component of IMCs phase, Yang et al. [10] added Zn element to the filler wire, and they suggested that the mechanical property of weld joint could be improved by a new phase of FeZn_10_ which has low brittleness and hardness.

Recent years, some studies reported that the morphology of the IMCs phase also have a significant influence on the mechanical property. It was reported [11,12] that the Fe_2_Al_5_ phase was more important than FeAl_3_ phase since the Fe_2_Al_5_ layer was thicker than FeAl_3_. The interface between Fe_2_Al_5_ phase and the steel substrate shows two different morphologies, including irregular interface and smooth interface. Several researches [13,14,15] have reported that some cracks were formed at the interface of Fe_2_Al_5_/steel in the service conditions. Cheng et al. [16] reported that the irregular interface could result in a stress concentrator which cause cracks. The morphology with a rough Fe_2_Al_5_/steel interface will crack more easily than a flat interface. In addition, adding Si and Cu elements into the molten pool also reduce the thickness and smooth the interface layer as reported by Yousaf et al. [17] and Awan et al. [18]

Understanding the formation mechanism, crystal structure, and evolution process of the Al-Fe IMCs will help to optimize the process parameters and realize a high mechanical bonding. As the main part of IMCs layer, the crystal structure and growth behavior of Fe_2_Al_5_ phase attracted the attention of researchers. Burkhardt et al. [19] revealed that there was a high vacancy concentration in the C-axis of Fe_2_Al_5_ crystal lattice. The high number of lattice defects will induce a high diffusion of atoms which lead to a rapidly growth in the C-axis. Naoi et al. [20] studied the growth behavior of Fe_2_Al_5_ layer using diffusion couple. They found that the volume diffusion controlled the growth of Fe_2_Al_5_ layer, and the interdiffusion coefficient of Fe_2_Al_5_ is more than two orders of magnitude larger than FeAl_3_ phase. Szczepaniak et al. [21] studied the interface thermals and the growth manner of Fe_2_Al_5_ layer, they suggested that the peak temperature has a stronger influence on the thickness of IMCs layer.

Previous studies show that the addition element and thermal characteristic had a great influence on the IMCs growth process and interface morphology [22]. However, most studies of IMCs growth remain in limited experimental observation and data. The mechanism of growth process, chemical reaction of Al and Fe atomic, and the atomic diffusion at the steel and aluminum dissimilar metals are still not completely understood. It is significant to understand the IMCs growth mechanism and phase evaluation, which can provide a foundation to optimum parameters and obtain a high mechanical bonding.

At present, the numerical models of the Monte Carlo method (MC), the cellular automata method (CA), and the phase field method (PF), which were used to describe the nucleation and growth of solidification microstructure. Many solidifications microstructure and phase transformation were carried out based on different simulation models. Zhang et al. [23] used the MC method to simulate the phase transform between α grain and β grain. Ogawa et al. [24] developed a CA model to describe the coarsening growth of γ grains. Yang et al. [25] established the multiple-physical CA to simulate the grain morphology and grain size in the electron beam welding process. However, using any single method or unmodified model has some shortcomings. Such as, the PF method has low computational efficiency since a large amount data need to be calculated. The MC model lacks physical basis and cannot quantitatively analyze physical phenomena. At present, improving the model or coupling multiple methods is an important means to solve the shortcomings of single model. This work proposed a novel model, which combined CA and MC simulation method, to simulate and predict the interfacial IMCs in the steel-aluminum welded joint.

In the present study, we focus on understanding the effects of thermal characteristic and elements on the IMCs layer growth manner by combining experiment and physical model. The microstructure morphology and phase identification of the Al-Fe IMCs layers were analyzed by electron backscatter diffraction (EBSD) and scanning electron microscopy (SEM). Phase chemical composition was identified by energy dispersive spectrometry (EDS). A numerical model of IMCs growth process was established using a Monte Carlo (MC) combined cellular automaton (CA) method, the growth behavior and microstructure morphology were studied. The relationship between IMCs thickness and process parameters also be analyzed.

## 2. Experimental Details, Materials and Model Establishment

### 2.1. Material and Characterization of Microstructures

All the specimens for microstructure analysis were cut from welded joint that fabricated by laser beam. The welding material are galvanized steel with the size of 100 mm × 50 mm × 1 mm and 5A06 aluminum alloy with the size of 100 mm × 50 mm × 2 mm, the ultimate tensile strength of aluminum and steel were 330 MPa and 290 MPa, respectively. The chemical compositions were listed in the Table 1, which taken from manufacturer data.

**Figure 1:**
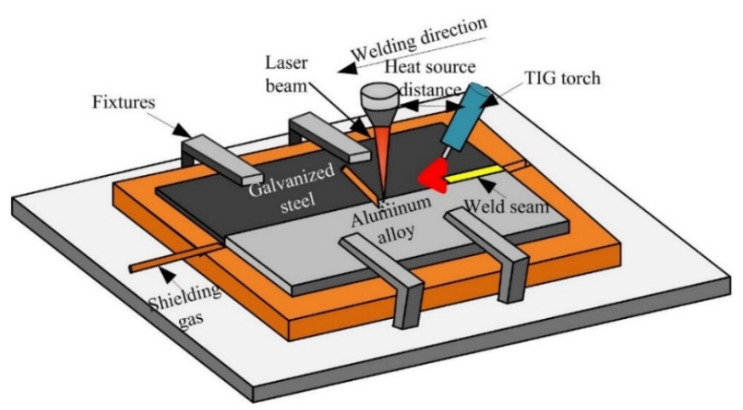
The schematic diagram of arc assisted laser welding-brazing.

The arc assisted laser welding-brazing method was applied to join steel and aluminum in a butt configuration [26]. Figure 1 shows the experiment system, in the welding process, the laser beam irradiation on the aluminum alloy base metal, and the angle between the TIG welding torch and base metal was 45° and the distance between the laser heat source and arc was 15 mm. The welding parameters were listed in the Table 2.

Specimens were cut from the beads along the direction perpendicular to the welding direction. The cross-section of the specimen was mechanically ground with abrasive papers and polished to obtain a mirror-like surface. Surface etching was performed with the Kellers etchant (5 mL HNO_3_, 3 mL HCl, 2 mL HF, and distilled water).

**Figure 2:**
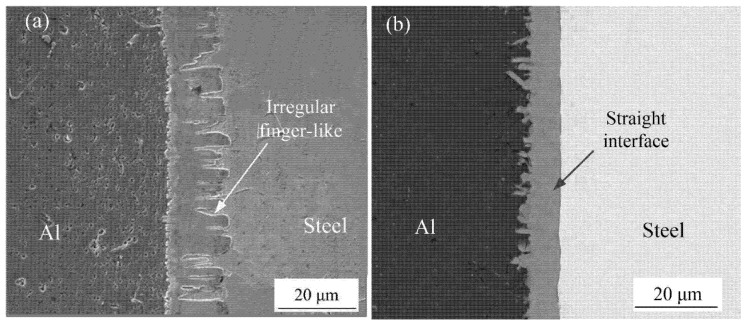
Interface morphology: (**a**) without adding Si element and (**b**) adding Si element.

The SEM was used to observe the microstructures of the Al-Fe interface, there were two different interfacial morphology between IMCs and steel with adding Si element or without adding Si element, as shown in Figure 1. Figure 2a shows the interfacial phase was obtained without adding Si element during welding process, the morphology of IMCs-steel interface showed an irregularly finger-like and grew to steel substructure. However, in the condition with adding Si element, it was found that the finger-like shape was disappeared and showed a smooth morphology, as shown in Figure 2b.

The energy dispersive spectrometry (EDS) was used to phase identified and analyze the chemical composition of the IMCs. The electron backscatter diffraction (EBSD) was used to analysis the crystal orientation and grain size of the IMCs phases. The coordinate system of EBSD stage was defined as RD-TD-ND, the normal ND indicated the direction perpendicular to the cross section of the weld seam, the transverse TD indicated the direction parallel to the surface of substrate, and the rolled RD indicated the direction parallel to the cross section of weld seam. Data and image were captured and analyzed by OIM software. During the test process, scan step size was 0.3 um. In order to improve the accuracy of test data, the grain boundaries of which orientation difference was less than 1°.

### 2.2. Numerical Modeling

**Figure 3:**
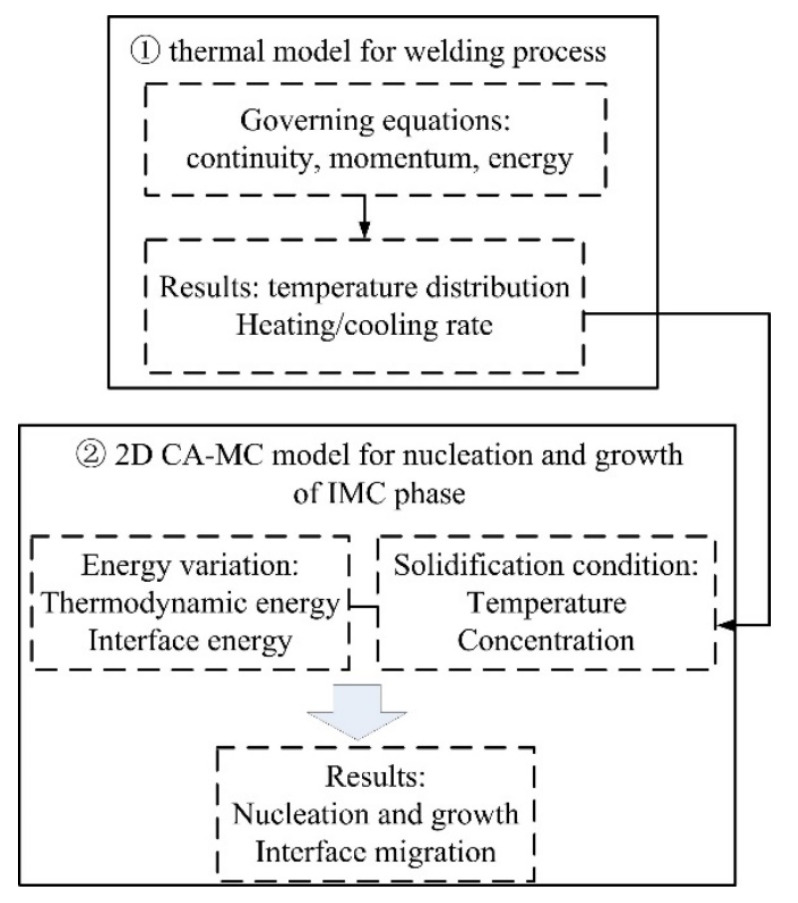
Flow chart of the CA-MC model.

A 3D thermal model was first performed to simulate the temperature distribution in the laser welding process of steel and aluminum. Then the interface thermal information was incorporated into the combined 2D CA-MC model, which includes energy variation and solidification condition, to simulate the IMCs grain morphology and growth characterization. A flow chart of the simulation model is shown in Figure 3.

**Figure 4:**
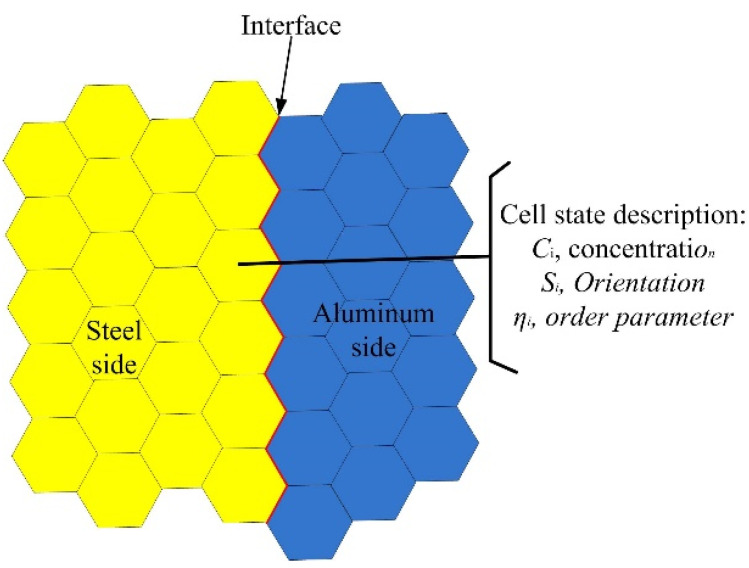
Schematic diagram of two-dimensional lattices.

The interfacial microstructures were mapped onto a 2D hexagonal lattice. The orientation, the Al/Fe concentration, and the order parameter were employed to describe the state of each hexagonal cell. Figure 4 shows the schematic diagram of lattices used in this model.

#### 2.2.1. Thermal Analysis

The nucleation and growth of IMCs is a diffusion-controlled process which are dependent on temperature and the diffusion of solute atoms [27,28,29]. A 3D finite element model of temperature field was developed by the authors’ research group to simulate the temperature distribution and thermal history curve at the interface during the laser welding process [26,30]. The model was successfully applied to simulate the temperature and residual stress in dissimilar welded joint. Due to the model being introduced in the authors’ previous publications, the governing equations and boundary conditions will not be presented in this work.

In the present study, to simplify the analysis and correspond to two-dimension cells, the heat conduction in the welding process was approximated as a two-dimensional problem. Due to the topic of this work is focused on the interface between steel and aluminum, the temperature variations near the Al-Fe interface were employed. The effect of edge heat conduction was assumed to be negligible. In the center of this 2D domain, an interface zone with dimension of 100 (X) × 100 (Y) μm^2^ was created to cover the entire interface zone.

#### 2.2.2. Energy Change

In the model, the total energy includes two components: the Gibbs energy term for Al-Fe phase and interface energy term. The total energy in the system can be calculated by following equation [31]:

(1) $$H=\frac{1}{2}\sum_{i}^{n}\sum_{j}^{m}E_{S_{i}S_{j}}^{}+\sum_{i}^{n}E_{i}^{}$$

where *S_i_* is the orientation of cell *i*, *S_j_* is the orientation of cell *j* that is the one of the nearest grain sites of cell *i*. $E_{S_{i}S_{j}}^{}$ is the interface energy between cell *i* and cell *j*. *E_i_* is the free chemical energy of site *i* with *S_i_* orientation. For the interface energy, the Read–Shockley model [32] is used to calculate the interface energy, it can be expressed by following equation:

(2) $$\gamma =\gamma_{m}(\frac{\theta }{\theta^{m}})[1-\ln(\frac{\theta }{\theta^{m}})]$$

where *θ* is the grain boundary of misorientation, *γ_m_* = 0.56 J·m^−2^ and *θ_m_* = 15° when the boundary becomes a high grain boundary.

For the free chemical energy, the Molar Gibbs free energy was adopted to calculate the system chemical energy. The pressure is equal to the standard atmosphere pressure during welding process; therefore, the Gibbs free energy of IMCs phase is a function of composition and temperature, it can be given as [33]:

(3) $$G_{m}^{\varphi }=G_{0}^{\varphi }+G_{mix}^{i}+G_{mix}^{ex}$$

where *φ* indicates possible phase, $G_{0}^{\varphi }$ is the mechanical mixture of pure components to the Gibbs energy, $G_{mix}^{i}$ is the ideal mixing contribution, and $G_{mix}^{ex}$ is the excess energy caused by the interaction of different composition. For a disordered binary phase, including solid solution phases or IMCs, the expression of each term in Equation (3) can be written as [33]:

(4) $$G_{0}^{\varphi }=\sum_{k=1}^{m}X_{k}G_{k}^{\varphi }$$

(5) $$G_{mix}^{i}=RT\sum_{k=1}^{m}X_{k}\ln X_{k}$$

(6) $$G_{mix}^{ex}=\sum_{k=1}^{m-1}\sum_{l=k+1}^{m}X_{k}X_{l}\sum_{n=0}^{m-1}[L_{kl,n}{\left(X_{k}-X_{l}\right)}^{n}]+G^{XS}$$

where *X_k_* is the molar fraction of composition k, *m* is the component number in the system, and $G^{XS}$ is the higher-order energy term in $G_{mix}^{ex}$. For a binary phase, $G^{XS}$ can be expressed by the following equation:

(7) GXS=X1X2Lter

The present study focuses on the formation and growth of the IMCs phase, which the crystal structure is a disordered structure. In this way, the standard solution model is used to calculate the molar Gibbs free energy for different IMCs phases:

(8) G0φ=XFeGFeφ+XAlGAlφ+RT(XFelnXFe+XAllnXAl)+XFeXAl[L0Al,Fe+L1Al,Fe(XAl−XFe)+L2Al,Fe(XAl+XFe)2]

The Gibbs energies of each component in liquid or in standard states can be found in the Scientific Group Thermodata Europe (SGTE) element database [34].

#### 2.2.3. Solute Distribution Calculation

The solute concentration in solid and liquid phases are governed by the diffusion equations for each phase [35]:

(9) $$\frac{\partial c}{\partial t}=\nabla \cdot (D\nabla c)$$

where *c* is the solute concentration and *D* is the iron diffusion coefficient in the Al liquid or Al diffusion coefficient in the IMCs.

#### 2.2.4. Nucleation and Growth

The classical nucleation theory is employed to express the nucleation rate of phases, as following equation [36]:

(10) $$I=I_{s}\exp(-\frac{\tau }{t})$$

where $\exp(-\frac{\tau }{t})$ is a time relate term and *I_s_* can be expressed by following equation [36]:

(11) $$I_{s}=KD_{r}{\left(kT\right)}^{-\frac{1}{2}}\exp(-\frac{K_{1}}{kT{\left(\Delta G_{v}\right)}^{2}})$$

where *K* is a constant expressed the nucleation density, *K*_1_ is a constant related to all the interfaces involved in nucleation, i.e., the steel/IMCs interface, *D_r_* is the diffusion coefficient, *k* is the Boltzmann’s constant, and $\Delta G_{v}$ is the driving force of volume change for nucleation process.

The crystal growth model in current work was followed the lowest energy principle. When a cell *i* was selected with a certain concentration, and meantime the nearest cell *j* and cell *k* were selected randomly. A new orientation will be appeared between two nearest cells, then the cell was given a new orientation and the boundary energy also changed. The total energy of the system before and after the above transition were calculated. The new orientation of the cell was adopted by comparing the difference system energy ΔE, which can be expressed by the following equation [36]:

(12) $$\Delta E=E_{1}-E_{0}$$

If Δ*E* ≤ 0, the new orientation of will be accredited. If Δ*E* > 0, all the attempts are accepted with a probability value as the following equation [36]:

(13) $$P=\exp(\frac{-\Delta E}{kT})$$

where *k* is the Boltzmann constant, *T* is the temperature value, and *P* is the probability of the new orientation.

## 3. Experimental and Simulation Results

### 3.1. Microstructure Analysis

**Figure 5:**
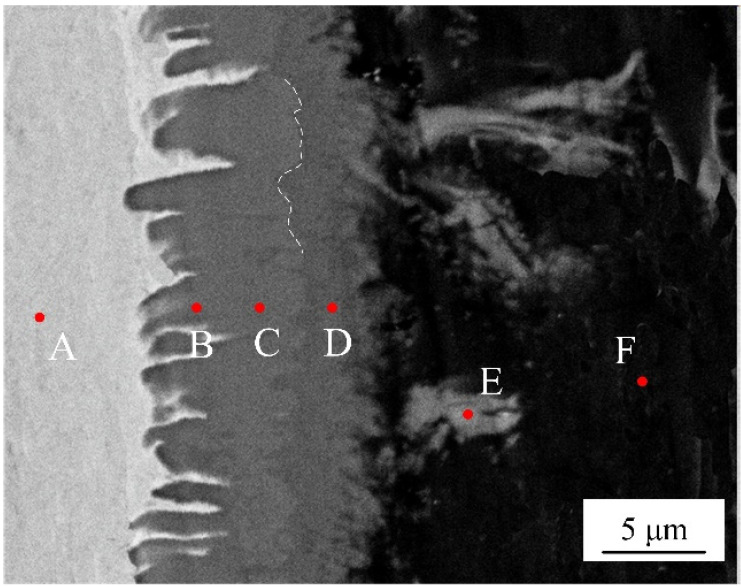
SEM image of selected locations (spots A~F) for EDS analysis.

EDS analysis was adopted to identify the phase chemical composition. Six spots were selected from different locations at interface without adding Si element, as shown in the Figure 5. The Table 3 list the chemical composition of selected test points. According to the atomic rate of Al to Fe from EDS results, the interfacial microstructures are composed of a layered needle-like Fe_2_Al_5_ phase and a flocculent-like Fe_4_Al_13_ phase. It is interesting to note that some flocculent-like structures were appeared in the aluminum substrate. According to EDS results and Fe-Al phase diagram [32], these structures may be the eutectic phase of Fe_4_Al_13_ and Al substrate.

**Figure 6:**
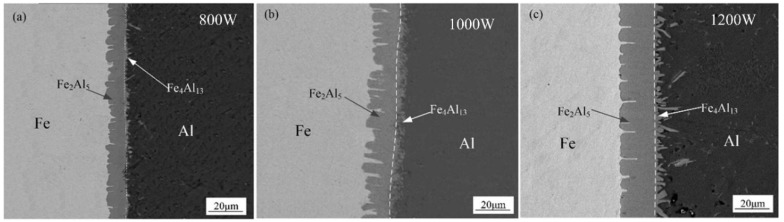
Interfacial micromorphology with different laser power: (**a**) 800 W, (**b**) 1000 W, and (**c**) 1200 W.

Figure 6 shows the SEM interfacial microscopic morphology without adding Si element. As shown in Figure 6, an intermetallic compound (IMCs) layer was formed between aluminum weld seam and steel, which present different morphologies near both sides of the substrate metals. The interface between steel substrate and IMCs layer shows a needle-like shape, and other interface between IMCs layer and aluminum is presented in a flocculent-like shape. With the increase of laser power, the flocculent-like grow into rod-like shape with random grain orientation. The thickness is increased with the increase of laser power.

**Figure 7:**
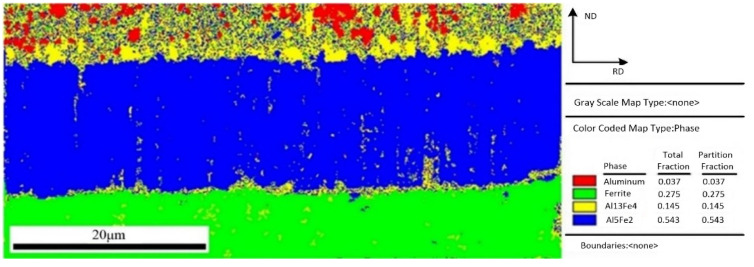
EBSD phase map of interface layer.

In order to get more understanding and characterize of the interfacial microstructure, the interfacial phase analysis between aluminum and steel was performed using electron backscattered diffraction (EBSD) method. The region with clear diffraction pattern and high calibration rate was selected to perform the EBSD map scanning. Figure 7 shows a EBSD phase color map of IMCs layer and the fraction of each phase. Four color regions with red, green, yellow, and blue depict four phases, in which these identified phases are Al, Fe, Fe_4_Al_13_, and Fe_2_Al_5_, respectively. As shown in Figure 7, the phase near the steel is Fe_2_Al_5_ phase, and another phase near the aluminum is Fe_4_Al_13_ phase. This result is agreement with the test conclusion of EDS in Figure 5. Comparing the area fraction of Fe_4_Al_13_ and Fe_2_Al_5_, it is obvious that Fe_2_Al_5_ has a larger fraction, which indicates that the IMCs formed in interface is mainly Fe_2_Al_5_.

**Figure 8:**
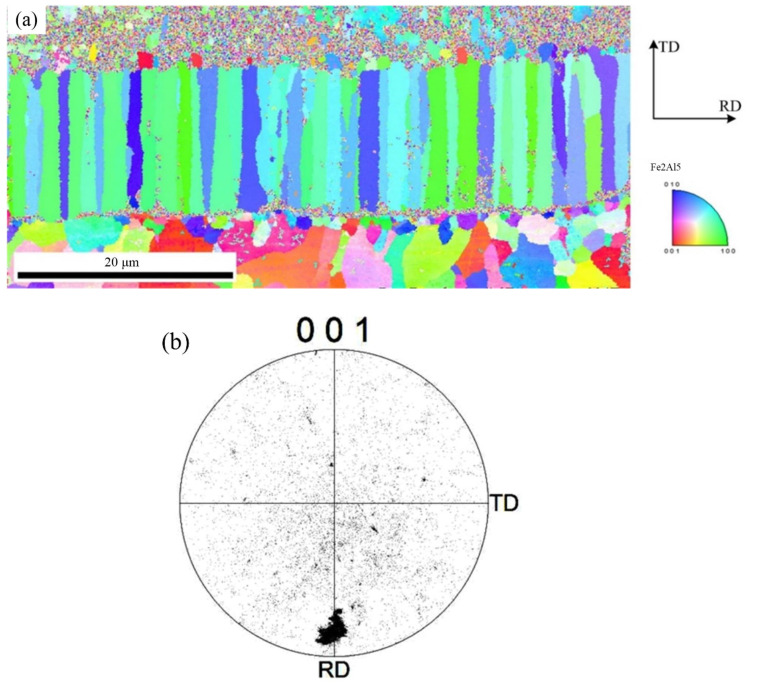
EBSD image of Fe_2_Al_5_ phase: (**a**) inverse pole figure map and (**b**) crystal orientation.

To further observe the distribution of Fe_2_Al_5_ phase and Fe_4_Al_13_ phase, the orientation mapping at the interface and the inverse pole figure in ND direction were analyzed, as shown in Figure 8. The crystal grain with same color indicates same orientation. Red of inverse pole figure indicates that the normal of crystalline grain is parallel to [001] direction, green indicates that the normal of crystalline grain is parallel to [001] direction, and blue indicates that the normal of crystalline grain is parallel to [010] direction. The phase morphology, grain size, and crystal orientation of different crystalline grains can be observed from Figure 8. Figure 8a indicates that the morphology of Fe_2_Al_5_ phase is the lath, and it belongs to large grain size. Figure 8b shows that the grain orientation of Fe_2_Al_5_ is mainly segregated at the bottom of the rolled RD, and is rarely distributed in other directions. The distribution of Fe_2_Al_5_ on (001) pole figure shows that the growth of lath Fe_2_Al_5_ has a directionally dependent property, which indicates that the crystal growth of Fe_2_Al_5_ phase is certain selectivity, and its distribution orientation is [001]. The growing of Fe_2_Al_5_ phase is along [001] direction preferentially.

**Figure 9:**
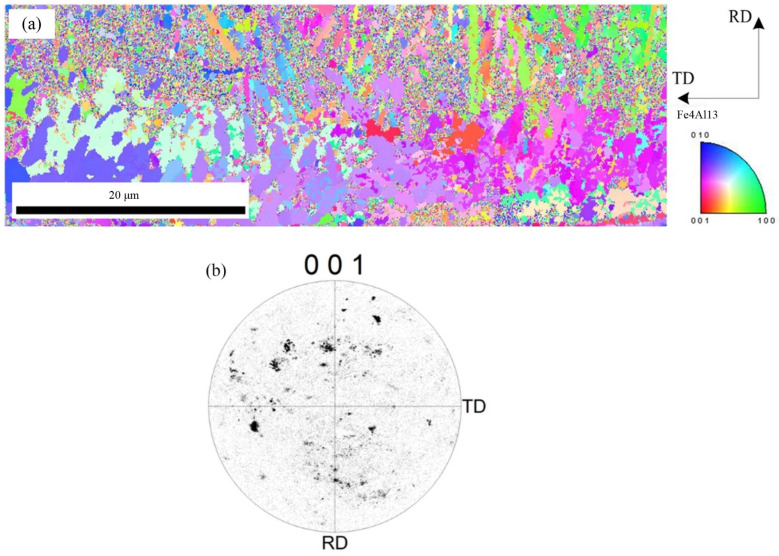
EBSD image of Fe_4_Al_13_ phase: (**a**) inverse pole figure map and (**b**) crystal orientation.

Figure 9 shows the microscopic orientation distribution and the [001] pole figure of Fe_4_Al_13_, respectively. Figure 9a indicates that the Fe_4_Al_13_ with flocculent or need-like morphology is growing from Fe_2_Al_5_ side to aluminum side. Compared with Fe_2_Al_5_ phase, the Fe_4_Al_13_ phase has a random distribution and a small grain size. As shown in the Figure 9b, the (001) pole figure of Fe_4_Al_13_ indicates that the Fe_4_Al_13_ phase is randomly distributed in different directions.

**Figure 10:**
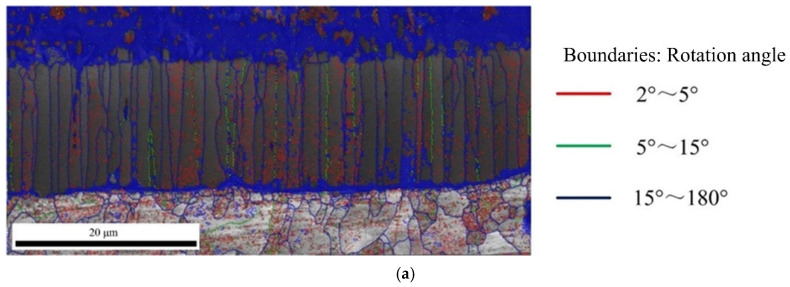

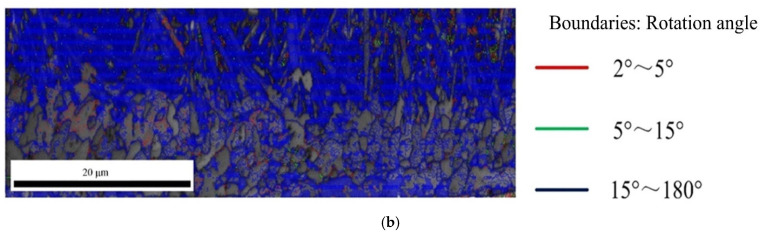
Angle distribution of grain boundary: (**a**) grain boundary in Fe_2_Al_5_ phase and (**b**) grain boundary in Fe_4_Al_13_ phase.

The Figure 10 shows the angle distribution of grain boundary in Fe_2_Al_5_ and Fe_4_Al_13_, red lines represent the misorientation (θ) of 2° < θ < 5°, green lines represent the misorientation (θ) of 5° < θ < 15°, and blue lines represent the misorientation (θ) of 15° < θ < 180°. In Figure 10a, it is worth to note that besides many large angle grain boundaries above 15° at the boundary of Fe_2_Al_5_, a larger number of small angle grain boundaries less than 5° was appeared inside Fe_2_Al_5_. As reported by Takata et al. [37], this phenomenon was caused by the transformation strain between α-Fe and Fe_2_Al_5_ phase during the solidification process. As shown in Figure 10b, the boundary and interior of Fe_4_Al_13_ grain contain a lot of large angle grain boundaries above 15°. Meanwhile, it is found that some small grain boundaries below 5° are appeared in a few grains.

**Figure 11:**
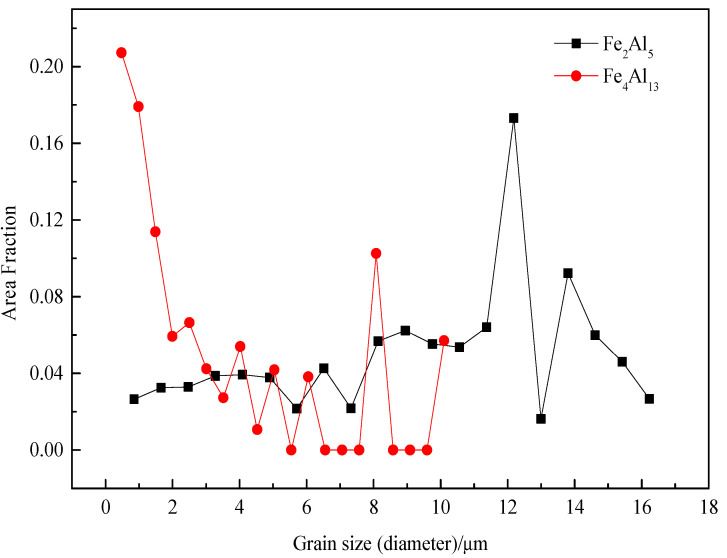
Grain size distributions of Fe_2_Al_5_ and Fe_4_Al_13_.

The grain sizes of Fe_2_Al_5_ and Fe_4_Al_13_ are statistically analyzed, as shown in Figure 11. All grains are smaller than 18 µm. The range of grain size of Fe_2_Al_5_ is from 0.8 µm to 16 µm, and the most area fraction of Fe_2_Al_5_ grain is about 12 µm. The grain size range of Fe_4_Al_13_ is from 0.5 µm to 10 µm, the most area fraction of grain is about 0.5 µm. The result suggests that Fe_2_Al_5_ phase is mainly consist of some large size grains and Fe_4_Al_13_ phase is mainly consist of many small size grains, which in agreement with the microscopic morphology in Figure 5.

### 3.2. Numerical Simulation of Al-Fe IMCs

**Figure 12:**
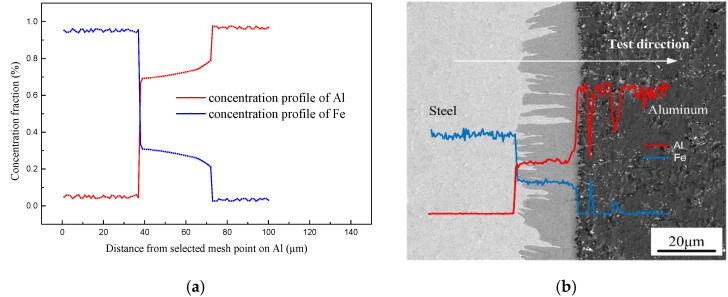
Concentration profiles of Fe and Al across interface: (**a**) solute distribution of simulated result and (**b**) EDS line scanning across the interface.

As reported by previous publications [38,39], the formation and growth of IMCs phases were dependent on local thermal characteristic and the change of the solute atom concentration. Figure 11 shows the concentration distribution of Al and Fe obtained by simulation result and EDS line scanning. The simulation has an approximate trend with EDS test result. As shown in the Figure 12a, the concentration profile of Al shows a stable stage in Fe_2_Al_5_ phase and an increased stage when closed to the Al side, which has an approximate trend with EDS test result shown in Figure 12b. The concentration of Al is almost approximately 72% which corresponds well to the EDS. It indicates that the simulation process of solution atom is basically consistent with the experiment, which suggests that the physical models used in the numerical calculation are reasonable.

**Figure 13:**
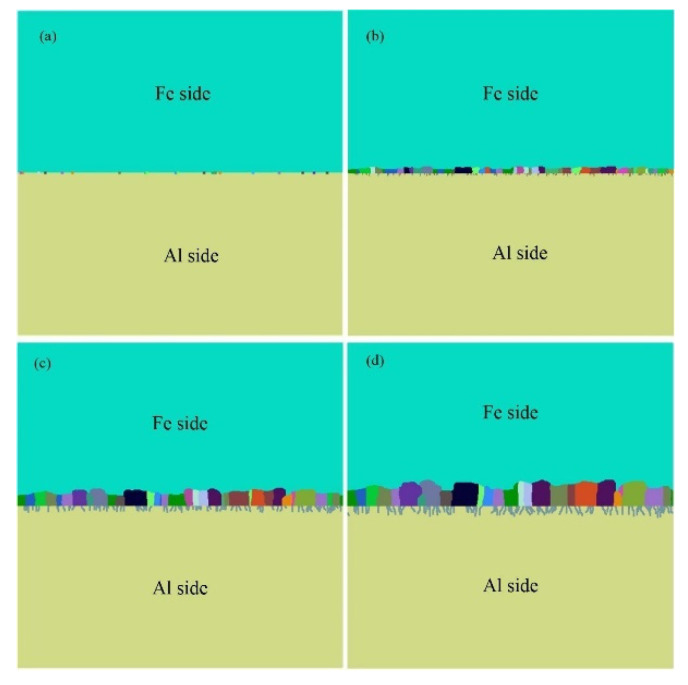
Simulated IMCs microstructure at different Monte Carlo steps: (**a**) 15,000 mcs, (**b**) 20,000 mcs, (**c**) 25,000 mcs, and (**d**) 30,000 mcs.

In the simulation model, 500 × 500 hexagonal meshes were used, and the length of the unit lattice was 0.2 μm. Figure 13 shows the simulated results of IMCs phase formation and growth process at different simulation times. At the early stage, there are some Fe_2_Al_5_ nucleation present at the Al/steel interface, which randomly distributed in the steel side, as shown in the Figure 12a. At second stage, the new nucleation and growing of IMCs occur at the same time, lead to form a layer of Fe_2_Al_5_ IMCs phase in the steel side, as shown in Figure 12b. From the Figure 13c, one can note that some new phases were appeared at the Fe_2_Al_5_/Al interface. It indicates that the Fe_4_Al_13_ IMCs formed in a lower temperature range, and the formation of Fe_4_Al_13_ may be caused by the reaction of Fe_2_Al_5_ + Al(liquid) → Fe_4_Al_13_. Figure 13d shows the solidification morphology of Al-Fe IMCs phases at the interface. This final IMCs layer contains double sublayer that are Fe_2_Al_5_ phase layer and Fe_4_Al_13_ phase layer, which is in accord with the experimental observation and some results reported in other publications [40,41,42]. On the other hand, the interfacial morphology of Fe_2_Al_5_/steel is displayed in a finger-like shape, and the interfacial morphology of Fe_4_Al_13_/Al is presented in a needle-like shape. In the simulation result, the Fe_2_Al_5_ phase grows in columnar structure and towards to the steel substrate, similar to the microstructures shown in the Figure 5.

**Figure 14:**
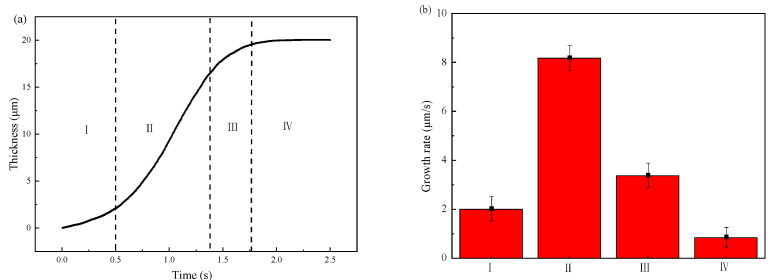
Variations of the IMCs thickness with the simulation time: (**a**) IMCs growth curve and different stages of I–IV, (**b**) growth rate at different stages.

Figure 14 shows the simulated results of the thickness variation and the growth rate of IMCs layer. From Figure 14a, one can divide the growth process into four stages according to the growth rate shown in Figure 14b. The first stage is nucleation of the IMCs phases which has a small growth rate since most of energy is consumed for nucleation. After nucleation process, the following is the second stage with the fastest growth rate of 8 μm/s. In this stage, the fast growing of IMCs layer is mainly controlled by vacancy diffusion of Al due to the high vacancy concentration along C axis in the Fe_2_Al_5_ phase [43]. Following the fast growth stage is the third stage with a slower growth rate than second stage. The lower growth rate in this stage may be explained by the long diffusion distance of Al atoms due to the formed Fe_2_Al_5_ phase in second stage. The last stage with the lowest growth rate. In the last stage, the growth rate is the lowest due to the decrease of local temperature which reduce the migration rate of Al atom. As shown in Figure 14b, one can sum that the growth rate of IMCs layer first increases with the simulation time increases, reaches the maximum, and then decreases to the lowest. It indicates that the diffusion condition is different during the growth process.

The thermal cycle plays an important role to determine the growth of IMCs layer. In order to obtain the correlation between thermal characteristic and IMCs growth, the effect of peak temperature, cooling time, and temperature gradient on the average thickness of Fe_2_Al_5_ phase were studied.

**Figure 15:**
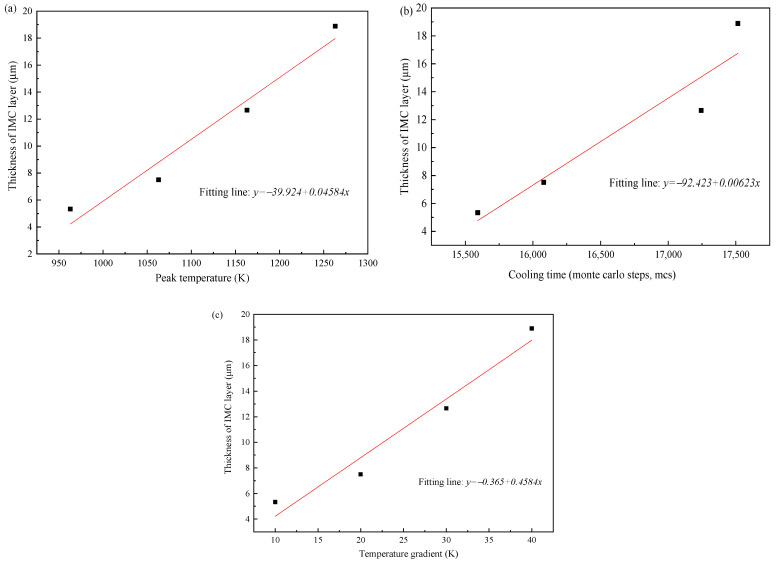
(**a**) Correlation between peak temperature and IMCs thickness, (**b**) correlation between cooling time and IMCs thickness, and (**c**) correlation between temperature gradient and IMCs thickness.

Figure 15 shows the relationship between the IMCs layer thickness and the three thermal factors, respectively. The IMCs thickness is increasing with the increase of three influence factors and shows a linear relationship. For the peak temperature shown in Figure 15a the average thickness of Fe_2_Al_5_ phase decreases significantly with the decrease of peak temperature. The reason is that the peak temperature has a great influence on the mutual diffusion between Al and Fe. The low peak temperature will slow the nucleation rate of Fe_2_Al_5_ and makes the Fe_2_Al_5_ layer not formed quickly. This restricts the rapid diffusion of Al and reduces the thickness of intermetallic compound layer. The temperature gradients were added to the direction of [001] surface of Fe_2_Al_5_. As shown in Figure 15c, the average IMCs thickness increases with the increase of temperature gradient, but the change trend is not small. The reason is that the temperature gradient in the direction of [001] only affects whether the Fe_2_Al_5_ tends to grow in the direction of [001]. When a great number of Fe_2_Al_5_ phases grow to the direction of [001], the grow in other directions is limited.

## 4. Discussion

This paper studies the interface microstructure of Al/Fe weld joint and mainly focus on the growth behavior of IMCs phase layer. The understanding of the growth mechanisms of Fe-Al phase and how to control the IMCs from process parameters are significant to improve mechanical property of Al-steel hybrid joint. Formation and growth process of IMCs layer, effect of thermal cycle on the thickness of IMCs layer, and the variations of interfacial morphology will be discussed in this part.

### 4.1. Formation and Growth Process of IMCs Phase

**Figure 16:**
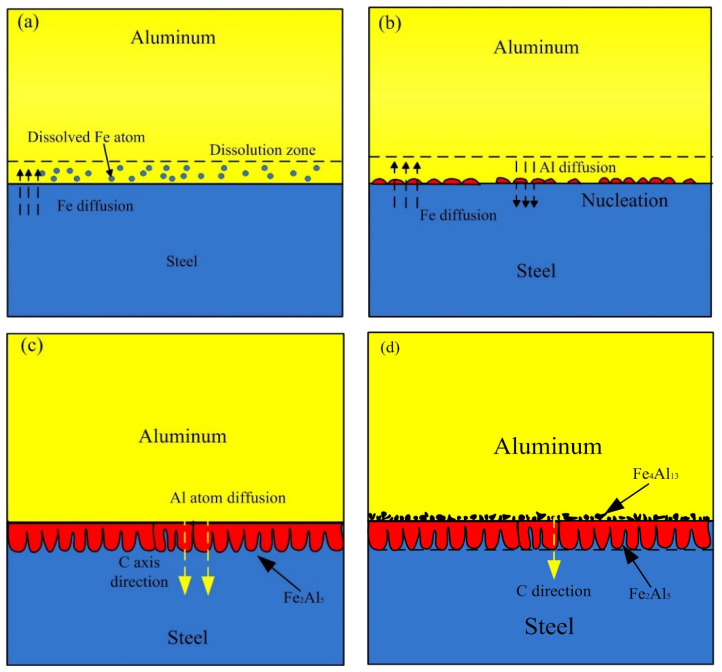
Schematic diagram of the formation and growth of the Al-Fe IMCs layer: (**a**) Fe atoms diffusion process, (**b**) nucleation process, (**c**) growth process and (**d**) final process.

Based on the experiment observation and the numerical model, the process of formation and growth of the IMCs phases at the Al-Fe interface was presented as shown in Figure 16. In the early stage, some Fe near the liquid Al is dissolved and diffuse into liquid Al, causing a dissolution zone which Fe atom enrichment in the liquid Al, as shown in Figure 16a. Figure 16b shows the second stage, the Fe_2_Al_5_ phase formed by the chemical reaction of Al atom and Fe atom at the boundary of liquid and solid. In this stage, the thin and random nucleation allowed the interdiffusion of Al and Fe atoms till the formation of Fe_2_Al_5_ phase layer which restrict the dissolution of steel. The energy consumption of this stage is nucleation of new phase, and the chemical reaction control the process. In the third stage as shown in Figure 16c, this stage is mainly the growing process of Fe_2_Al_5_, and it grows, preferably, along the C-axis in the crystal lattice. There are 30% of the vacancies along the c-axis in the Fe_2_Al_5_ crystalline lattice, which proposals a rapid diffusion path for Al atoms. The faster diffusion rate of Al atoms along C-axis direction in Fe_2_Al_5_ makes the interface morphology of steel-Fe_2_Al_5_ appear a discontinuous tongue-like shape. This growth morphology is also observed by Krisam et al. [44], and can be described by above simulation model. The last stage is illustrated in the Figure 16d, the solubility of Fe in Al molten decreased due to the cooling process, then the Fe_4_Al_13_ phase formed adjacent to Fe_2_Al_5_ phase through chemical reaction. This stage indicates that the Fe_2_Al_5_ phase was formed prior to Fe_4_Al_13_ phase, it can be explained by the Gibbs free energy change of two different phases. As reported by Wang et al. [45], the Gibbs free energy of Fe_2_Al_5_ phase is more negative than that of Fe_4_Al_13_ phase. It suggests that the formation reaction of Fe_2_Al_5_ is easier than Fe_4_Al_13_.

### 4.2. The Effect of Thermal Cycle Characteristic on the Growth of IMCs Layer

**Figure 17:**
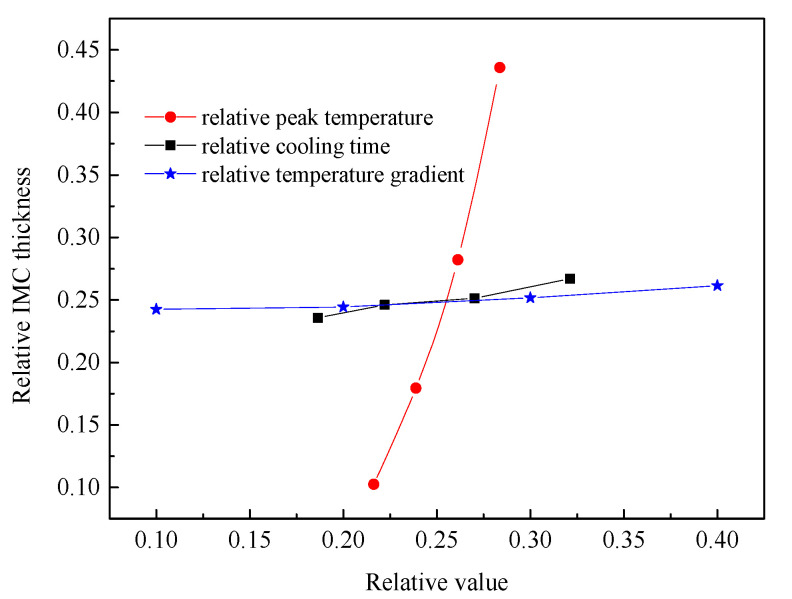
Correlations between the thicknesses of the respective IMCs layers and the peak temperature, cooling time, and temperature gradient.

Based on the above simulation results, there are three thermal factors influence the IMCs layer thickness. The effects of peak temperature, temperature gradient, and cooling time on the Fe_2_Al_5_ phase thickness were analyzed at the same time. Meanwhile, the cooling time, peak temperature, temperature gradient, and growth thickness were normalized by the respective maximum occurring values, and the comparison of the different condition parameters regarding their respective influence on the IMCs layer thickness result is shown in Figure 17. These results indicate that the sensitivity of Fe_2_Al_5_ phase thickness to peak temperature is much higher than that of the temperature gradient and cooling time by comparing the rising trend. In other word, the welding power plays an important role to control the over thick of the IMCs. To control the growth of the Al-Fe IMCs, one can optimize the welding power first rather than welding speed.

### 4.3. The Effect of Si Element on the Growth of IMCs

As observed in the Figure 2, the interface morphology of Fe_2_Al_5_-steel showed a flat shape for adding Si element, while the serration-shape was appeared without adding Si element. Once a continuous layer was formed, the following growth required diffusion of the Al and Fe atom through the IMCs layer. The crystal structure of the IMCs grain has a key effect on diffusion rate through the layer. The formation of tongue-like shape can be explained by high diffusion rate along C-axis since the high vacancy concentration in the C-axis direction. It is assumed that the Si atoms occupy the vacancy position first that will limit the C-axis diffusion of Al atom.

**Figure 18:**
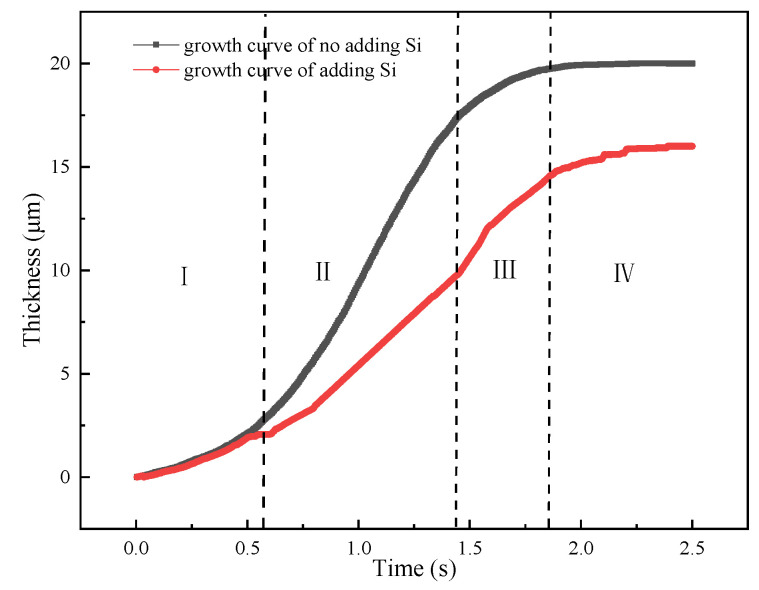
IMCs growth curves of adding Si element and without adding Si element and different stages of I–IV.

As previous studies, the most significant influence of Si addition on IMCs growth was to reduce the activation energy and diffusion coefficient. In the simulation model, the diffusion coefficient was reset as 0.5 m^2^/s [46] when Si element was added. Figure 18 shows the growth curves of adding Si element and without adding Si element. It can be from figure that the growth curves of two cases present a “S” tend. However, the growth rate of adding Si element is higher than that of no adding Si element, especially in the second stage. This calculated result might indicate that the diffusion path has changed from vacancy diffusion to grain boundary diffusion that will reduce the diffusion rate and growth thickness.

## 5. Conclusions

In the present study, the microstructure characteristics and phase identification of IMCs layer were investigated, the growth behavior of Fe_2_Al_5_ phase was discussed by physical model and experiment method. Major conclusions of this study could be summarized as followings:

(1) An IMCs layer compose of Fe_2_Al_5_ phase and Fe_4_Al_13_ phase were formed at Al/Fe interface. As the main phase Fe_2_Al_5_ grows along [001] direction. The maximum thickness of IMCs layer was 22 ± 2 μm for the laser power of 1200 W. The addition of Si element will reduce the IMCs thickness and change the interfacial morphology of steel/Fe_2_Al_5_.
(2) The IMCs growth process was simulated by combined MC/CA method. The growth process can be divided into four stages: the nucleation stage with the lowest growth rate, the diffusion growth stage that shows the fastest growth with the growth rate of 8 μm/s and determines the IMCs thickness, the long-distance diffusion stage with lower growth rate, and the stable stage.
(3) The effect of thermal characteristic on the thickness of Fe_2_Al_5_ IMCs phase is discussed, the sensitivity of Fe_2_Al_5_ phase thickness to peak temperature is much higher than that of the temperature gradient and cooling time. The addition of Si elements will influence the second growth stage of IMCs and limit the excessive growth of IMCs phase.

## Figures and Tables

**Table 1:** Chemical composition of base materials.

Elements	Si	Mg	Mn	Cu	Zn	Fe	P	S	Ni	Al
5A06 aluminum	0.4	5.8–6.8	0.5–0.8	0.10	0.2	0.40	-	-	-	Bal.
Galvanized steel	≤0.40	-	0.40	≤0.15	≤0.15	Bal.	0.02	≤0.30	≤0.15	-

**Table 2:** Welding parameters.

Welding Parameters	Laser Power (W)	Arc Current (A)	Heat Source Distance (mm)	Traveling Speed (mm/s)
Sample no. 1	800	15	15	10
Sample no. 2	1000	15	15	10
Sample no. 3	1200	15	15	10

**Table 3:** Chemical composition of selecting spots in Figure 4.

Spot	Al (at. %)	Fe (at. %)	Possible Phase
A	0	100	Fe
B	72.24	27.76	Fe_2_Al_5_
C	73.89	26.11	Fe_2_Al_5_
D	77.32	22.68	Fe_4_Al_13_
E	78.63	21.37	Fe_4_Al_13_
F	99.68	0.32	Al
